# Supplementary material for: Recruitment of two dyneins to an mRNA-dependent Bicaudal D transport complex
Source: eLife. 2018 Jun 26;7:e36306. doi: 10.7554/eLife.36306 (PMC6056235; doi:10.7554/eLife.36306)
Supplement: Transparent reporting form [file elife-36306-transrepform.docx]

***eLife’s* transparent reporting form**

We encourage authors to provide detailed information *within their submission* to facilitate the interpretation and replication of experiments. Authors can upload supporting documentation to indicate the use of appropriate reporting guidelines for health-related research (see [EQUATOR Network](http://www.equator-network.org/%20)), life science research (see the [BioSharing Information Resource](https://biosharing.org/)), or the [ARRIVE guidelines](http://www.plosbiology.org/article/info:doi/10.1371/journal.pbio.1000412) for reporting work involving animal research. Where applicable, authors should refer to any relevant reporting standards documents in this form.

If you have any questions, please consult our Journal Policies and/or contact us: [editorial@elifesciences.org](mailto:editorial@elifesciences.org).

**Sample-size estimation**

- You should state whether an appropriate sample size was computed when the study was being designed
- You should state the statistical method of sample size computation and any required assumptions
- If no explicit power analysis was used, you should describe how you decided what sample (replicate) size (number) to use

Please outline where this information can be found within the submission (e.g., sections or figure legends), or explain why this information doesn’t apply to your submission:

Sample sizes were not computed prior to the start of the experiments. Sample sizes are in accord or exceed the standard typically obtained in single molecule experiments.

**Replicates**

- You should report how often each experiment was performed
- You should include a definition of biological versus technical replication
- The data obtained should be provided and sufficient information should be provided to indicate the number of independent biological and/or technical replicates
- If you encountered any outliers, you should describe how these were handled
- Criteria for exclusion/inclusion of data should be clearly stated
- High-throughput sequence data should be uploaded before submission, with a private link for reviewers provided (these are available from both GEO and ArrayExpress)

Please outline where this information can be found within the submission (e.g., sections or figure legends), or explain why this information doesn’t apply to your submission:

Single filament TIRF assay experiments were repeated:

- 3 times with two independent preparations of dynein-dynactin for Figure 1D, Drosophila BicD^CC1^(Figure 2B,C), 2 independent experiments
- 3 times with one preparation of dynein-dynactin for the single molecule pull-down experiments (Figure 4A-C). Heterologous mRNAs and control (Figure 4C) 2 independent experiments.
- 3 times with one preparation of dynein-dynactin for Egl-K10 mRNA dual imaging (Figure 4E)
- Overall 4 times with two independent preparations of dynein-dynactin for mRNA motility experiments (Figure 5). Speed and run length of DD(BicDN) versus DDBE+K10 mRNA were directly compared 2 independent times, each pair on the same day with the same dynein-dynactin preparation. Drosophila DDB^CC1^ versus DDBE+K10 mRNA were directly compared 2 independent times.
- Once with multiple flow cells with one preparation of dynein-dynactin for the dual labeled Egalitarian experiment (Figure 6)
- 3 times with two independent preparations of dynein-dynactin for recruitment of two dynein motors to the mRNP using Alexa dyes (Figure 7A,B). Percentage of dual color complexes (yellow) were counted manually.
- 2 times with two independent preparations of dynein-dynactin for recruitment of two dynein motors to the mRNP using Qdot labeling (see Video 2 and Figure 7C,D).
- Figure 8 with or without LC8 3 independent experiments.
- Figure 9 two mRNA experiment 6 independent experiments.
- Figure 10 zippered Egl 5 independent experiments.
- Run lengths less than 300 nm were ignored.
- For speed analysis, clearly visible diffusional components (if any) were ignored during analysis.
- Experiments were carried out over the course of 3 years.

For electron microscopy:

Measurements were carried out on images in which the feature of interest could be clearly discerned. Sample sizes for all measurements conducted on electron microscopy data are reported in the corresponding results section. Calculated errors are reported as standard deviations alongside the mean values.

The global average and variance images shown in Figure 2C are the result of aligning and averaging 4205 particles of BicD selected from 126 micrographs. The b-type average and variance images shown in Figure 2C are the result of aligning a 2471 subset of this data containing only the most common b-type images. The subset was obtained by manual selection of b-type classes from an initial classification of the whole dataset into 20 classes.

The b-type average of BicD-Egl was obtained by initial alignment of 4117 particles from 98 micrographs of the complex, classification into 20 classes and manual selection of the most common b-type classes to create the b-type subset. This subset of 2484 images was aligned and averaged to produce the image shown in Figure 3B. The subtraction image shown in Figure 3C results from combination of the BicD b-type dataset with the BicD-Egl b-type dataset. The combined dataset was aligned and Figure 3C shows the result of subtraction of the average of the BicD dataset from the average of the BicD-Egl dataset.

The Egl position heatmap shown in Figure ure 3C results from marking the position of the additional mass assumed to be Egl in 342 aligned images of BicD-Egl in which the additional mass could be clearly discerned.

**Statistical reporting**

- Statistical analysis methods should be described and justified
- Raw data should be presented in figures whenever informative to do so (typically when N per group is less than 10)
- For each experiment, you should identify the statistical tests used, exact values of N, definitions of center, methods of multiple test correction, and dispersion and precision measures (e.g., mean, median, SD, SEM, confidence intervals; and, for the major substantive results, a measure of effect size (e.g., Pearson's r, Cohen's d)
- Report exact p-values wherever possible alongside the summary statistics and 95% confidence intervals. These should be reported for all key questions and not only when the p-value is less than 0.05.

Please outline where this information can be found within the submission (e.g., sections or figure legends), or explain why this information doesn’t apply to your submission:

Standard errors of the mean for single molecule pull-downs (Figure 1 C, 1D, 4 B). Standard errors of the mean for motility frequency (Figure 4 C). Standard errors of the mean were computed for each run length, and standard deviations were computed for speed using Graph Pad Prism software. Statistical differences between run lengths were calculated using the Kolmogorov–Smirnov test (Figure 5C, 5E, 6D, 7E, 8B, 9E, 10D). Statistical differences between speed distributions were calculated using a t-test (Figure 5B, 5D, 8A). n and p values are shown in the figure legends. p-values less than 0.05 were considered as significantly different.

(For large datasets, or papers with a very large number of statistical tests, you may upload a single table file with tests, Ns, etc., with reference to sections in the manuscript.)

**Group allocation**

- Indicate how samples were allocated into experimental groups (in the case of clinical studies, please specify allocation to treatment method); if randomization was used, please also state if restricted randomization was applied
- Indicate if masking was used during group allocation, data collection and/or data analysis

Please outline where this information can be found within the submission (e.g., sections or figure legends), or explain why this information doesn’t apply to your submission:

N/A

**Additional data files (“source data”)**

- We encourage you to upload relevant additional data files, such as numerical data that are represented as a graph in a figure, or as a summary table
- Where provided, these should be in the most useful format, and they can be uploaded as “Source data” files linked to a main figure or table
- Include model definition files including the full list of parameters used
- Include code used for data analysis (e.g., R, MatLab)
- Avoid stating that data files are “available upon request”

Please indicate the figures or tables for which source data files have been provided:

N/A
